# The Journal’s Prosperity

**Published:** 1861-02

**Authors:** 


**THE JOURNAL'S PROSPERITY.**—We forgot to ask our subscribers at the close of the year to renew their subscriptions, and nearly every one of our exchanges failed to make any mention of the fact that it was a very good time for any of their subscribers who wanted to live a hundred years to order that very useful publication, HALL'S JOURNAL OF HEALTH, published at 42 Irving Place, New-York City, for only one dollar a year. But in spite of these omissions and the difficulties of the times, and another fact that we made no promises of the great things we were going to do, nor offered pictures as big as a barn-door, and as beautiful, as extra inducements, our subscription-list this first week in January is nearly four times as great as at the same time of any year since our commencement. We do not know of one delinquent subscriber for 1860, nor will there be for 1861. Crediting demoralizes those credited, and bankrupts worthy publishers in multitudes of cases.

